# Active listing and more consultations in primary care are associated with reduced hospitalisation in a Swedish population

**DOI:** 10.1186/s12913-018-2908-1

**Published:** 2018-02-09

**Authors:** Karin Ranstad, Patrik Midlöv, Anders Halling

**Affiliations:** 1 Department of Clinical Sciences in Malmö, Centre for Primary Health Care Research, Clinical Research Centre (CRC), Lund University, Skåne University Hospital, Jan Waldenströms gata 35, 205 02 Malmö, Sweden; 2 Nättraby Primary Health Care Centre, Nättraby, Sweden

**Keywords:** Primary health care, Hospitalisation, Multimorbidity, Socioeconomic status

## Abstract

**Background:**

Healthcare systems are complex networks where relationships affect outcomes. The importance of primary care increases while health care acknowledges multimorbidity, the impact of combinations of different diseases in one person. Active listing and consultations in primary care could be used as proxies of the relationships between patients and primary care. Our objective was to study hospitalisation as an outcome of primary care, exploring the associations with active listing, number of consultations in primary care and two groups of practices, while taking socioeconomic status and morbidity burden into account.

**Methods:**

A cross-sectional study using zero-inflated negative binomial regression to estimate odds of any hospital admission and mean number of days hospitalised for the population over 15 years (*N* = 123,168) in the Swedish county of Blekinge during 2007. Explanatory factors were listed as active or passive in primary care, number of consultations in primary care and primary care practices grouped according to ownership. The models were adjusted for sex, age, disposable income, education level and multimorbidity level.

**Results:**

Mean days hospitalised was 0.94 (95%CI 0.90–0.99) for actively listed and 1.32 (95%CI 1.24–1.40) for passively listed. For patients with 0–1 consultation in primary care mean days hospitalised was 1.21 (95%CI 1.13–1.29) compared to 0.77 (95%CI 0.66–0.87) days for patients with 6–7 consultations. Mean days hospitalised was 1.22 (95%CI 1.16–1.28) for listed in private primary care and 0.98 (95%CI 0.94–1.01) for listed in public primary care, with odds for hospital admission 0.51 (95%CI 0.39–0.63) for public primary care compared to private primary care.

**Conclusions:**

Active listing and more consultations in primary care are both associated with reduced mean days hospitalised, when adjusting for socioeconomic status and multimorbidity level.

Different odds of any hospitalisation give a difference in mean days hospitalised associated with type of primary care practice.

To promote well performing primary care to maintain good relationships with patients could reduce mean days hospitalised.

## Key points

Relationships with primary care could be associated with hospitalisation within the complex networks constituting healthcare systems.

Active listing and more consultations are associated with reduced hospitalisation, and difference within primary care shown.

Promoting well performing primary care practices and their relationship with patients could be an option to reduce hospitalisation.

## Background

The need for hospitalisation is expected to increase with severe morbidity, but is also related to individuals, local healthcare and society. Relationships are the central unit of analysis within the complex networks comprising healthcare systems [1]. Good relations between individuals in a population and well performing primary care has been shown to contribute to more adequate care, trust and better health [2, 3]. Patients’ attachment to and satisfaction with primary care are linked to their choice of primary care [4, 5]. To patients this is a complex choice related to trust [6, 7]. Socioeconomic status affect both individual morbidity and trust in health care [8–10]. Continuity of care from a primary care provider has impact on hospitalisation [11, 12].

Recognising primary care as a part of complex healthcare systems, hospitalisation could be analysed as an outcome of relationships in primary care [1]. Primary care could be described as a multidimensional care system, comprising structure and processes generating outcomes like quality, efficiency and equity [13]. Well performing primary care is characterised by a combination of person-focused care over time, use as first contact in health care, completeness of services and coordination of care [13]. Efficiency of care, such as reducing hospitalisation, is a primary care outcome [13, 14].

Several definitions of morbidity are associated with clinical management and health outcomes. Multiple diseases within the same person are acknowledged using comorbidity or multimorbidity. Sets of disorders are often used to study potentially avoidable hospital admissions [15, 16]. Morbidity burden is the overall impact of the different diseases in one person taking into account their severity [9, 10]. To analyse hospitalisation as an outcome of relationships with primary care a multimorbidity measure that allows for comparison between groups of patients with the same need for care, despite different patterns of disorders was considered most relevant [17].

In Sweden, primary care practices comprise general practitioners (GPs) organised within multidisciplinary teams. Listing in primary care was introduced to empower patients and to introduce market models. County councils regulate local health care, organising primary care in several quasi-market models, mandatory since 2010 [18]. Blekinge County Council introduced listing in primary care in 2004. Listing was mandatory passive or active and individual. Active listing gave no favours compared to passive listing to patients or practices, but might be considered an act of the patient to protect their relationship with a primary care practice. Number of consultations in primary care quantifies other aspects of the relationship between population and primary care.

How relationships with primary care, i.e. active listing and number of consultations, could affect hospitalisation while accounting for socioeconomic status and morbidity burden, have not previously been studied. Prior studies have used reported data from small samples analysing whether or not patients were hospitalised [2, 5, 12]. We combined individual data on socioeconomic status with hospitalisation and morbidity burden from patient records for a population within the same healthcare system. The aim was to study hospitalisation, i.e. odds of any hospital admission and mean days hospitalised, as an outcome of relationships with primary care when adjusting for the overall impact of sex, age, socioeconomic status and morbidity burden. The additional aim was to analyse whether there was any difference within primary care.

## Methods

### Study population and settings

The year 2007 represents a period with stability in regulations, funding and workforce settings in primary care in Blekinge. On 31 December 2007 the county of Blekinge had 151,731 inhabitants. Of these, 50.5% were men, and the average age was 42.7 years [19]. Health care was provided by two hospitals, five psychiatric clinics and 25 primary care practices. Half of the primary care practices were privately owned, and were established in all municipalities. A total of 65% were actively listed, ranging between 50 and 85% according to municipality. A majority (84%) were listed in practices owned by the county council. The percentage of any hospitalisaton within this population was 8.7% and mean days hospitalised was 0.89.

Information on education level or residence was missing for 3471 and socioeconomic data for individuals < 16 years of age for 24,741. We therefore restricted this study to the remaining 123,168 individuals. This population had an average age of 50.1 years and 50.2% were men. A total of 68% were actively listed and 83% listed with practices owned by the county council. The share of actively listed ranged from 56 to 86% according to municipality. The study population had a hospitalisation rate of 9.6% and mean days hospitalised was 1.0.

Listing status and mean consultations were used as proxies of aspects of relationships with primary care. Listing was mandatory passive at the nearest primary care practice. Patients initiated change to active listing at will, at the same or another practice within the county. Family members over 15 years of age made their choice separately, and active listing could be changed at will. The practice of choice administrated active listing. Patients or practices gained no obvious favours from primary care by active instead of passive listing. Primary care practices were obliged to accept any patient and to distribute care according to medical need. Under these circumstances, active listing could be seen as patients acting to protect their relationship with primary care.

Number of consultations in primary care balances demand for health care against availability and need for care. Mean number of consultations in primary care was 0.9 and in all healthcare 2.0. The share of more than 5 consultations in primary care was 1.8% and in all healthcare 10%. Adjusting for morbidity burden, more than mean number of consultations could be used as a proxy of having, or searching for, a relationship with primary care.

Practices in primary care could be public, owned by the county council, or private. Public practices were typically older, with more listed patients and GPs, than private practices. The county council contracted all primary care practices. This gave equal funding and regulations, but different settings and processes amongst primary care practices. Of patients listed in private primary care 60% had little or no need for health care, compared to 35% in public primary care; income and education were equally distributed.

Local government area, municipality, was the available factor relating to local society and geographic location. Municipality is also correlated with active listing [20, 21].

### Design

We performed a cross-sectional population-based study on hospitalisation as an outcome of primary care. Listing status and number of consultations were used as proxies of relationships with primary care, and the difference between two types of practices was investigated. We adjusted for sex, age, socioeconomic status and multimorbidity burden. Data on an individual level collected from electronic patient records and Statistics Sweden during year 2007 was used. This study was approved by the Regional Ethical Review Board at Lund University (application no. 2016/71). According to this approval, the study population was given the possibility to opt out from the study.

### Outcome

Odds of any hospital admission for the population of Blekinge County during year 2007 were estimated.

Mean days hospitalised for the population were estimated from odds of ever being admitted to hospital and mean number of days hospitalised if at risk of hospitalisation, in all health care (somatic and psychiatric hospitalisation) in Blekinge County during year 2007.

### Explanatory factors

Actively or passively listed at primary care practice, not listed was not an option. Active listing was considered a measure of good relationship in primary care.

Number of consultations with a doctor in primary care (GP) was categorised into six groups (0–1, 2–3, 4–5, 6–7, 8–9, 10-), and used to quantify other aspects of the patient-professional relationship in primary care. More than one consultation considered to be associated with having a relationship with primary care.

Primary care practices grouped in private (type A) and public (type B) according to ownership. The two categories also included other differences as size and time since establishment.

Sex and age, where age was grouped into 16–19, 20–39, 40–59, 60–79, 80- years.

Individual disposable income was categorised into four equally sized levels (quartiles).

Individual education was divided into four levels: 1) less than 9 years of education, 2) completed 9 years of compulsory education, 3) college degree, or 4) university degree.

Multimorbidity level, as a summary measure of morbidity burden, was calculated from patient records from all health care for 2007 using the Johns Hopkins Adjusted Clinical Groups Case Mix System (ACG). This is one of the summary measures aiming to link all diagnoses with need of healthcare, focussing on stratification of patients into groups according to diseases and conditions, age and sex. ACG weights patients’ diagnoses according to five clinical dimensions: duration, severity, diagnostic certainty, aetiology and need for specialist care into almost 100 mutually exclusive ACGs. Then ACGs are categorised into six multimorbidity levels with similar impact on need for healthcare despite different patterns of diagnoses. These multimorbidity levels are called Resource Utilization Bands (RUBs) and range from 0 (no need for health care) to 5 (very strong need for health care) [17, 22].

Multivariate statistical models were clustered on municipality (local government area, in order to account for covariation associated with local society). The five municipalities range from 10,849–49,931 inhabitants.

### Statistical analysis

Statistical analyses were performed with STATA version 14.1 (Stata Corporation, Texas, USA). Associations between variables were studied using the Pearson correlation.

Coefficient, a linear correlation of 0.2 was considered meaningful. Number of days hospitalised was found to be skewed, non-normally distributed with over-representation of persons without need for hospitalisation. Count data models were tested, and a clustered zero-inflated negative binomial model considered most valid. This is a combined model using a binary method to assess odds ratios (OR) of being at risk, here a logit model. Incidence rate ratio (IRR) was used to show the influence of increasing the explanatory factors in the negative binomial part of the model by one unit if at risk. Mean days hospitalised for the population were calculated as average marginal effects combining the logit and negative binomial parts of the model. The multivariate statistical model was adjusted for sex, age, socioeconomic status and multimorbidity level, the same set of variables were used for both parts of the model, and the model was clustered on municipality [23].

Model performance was assessed using several different methods. Akaike’s Information Criterion (AIC) has a penalty term for additional parameters, lower values preferred. Likelihood ratio statistics (LR test) test differences between nested models. Higher values indicate greater difference from the simpler model than lower values. Coefficient of variance (CV) standardises standard deviation, using absolute mean to allow for comparison of variance across models. C-statistics (AUC), equivalent to the area under the receiver operating characteristics (ROC) curve, 1 indicating perfect discrimination and 0.5 equal to chance.

## Results

### Descriptive statistics

During 2007 the percentage hospitalised during year 2007 was 9.6% and mean hospitalisation was 1.0 day. Total hospitalisation was 123,690 days. The 68% actively listed accounted for 77% of those admitted to hospital and 79% of days hospitalised. The 22% with more than two consultations in primary care accounted for 37% of those admitted to hospital and 39% of days hospitalised. The 83% listed in public primary care accounted for 86% of those admitted to hospital and 87% of days hospitalised (Table 1).

**Table 1:** Descriptive statistics for the population > 15 years of age in Blekinge in 2007 (*N* = 123,168)

Descriptive 2007 Population > 15 years	Group size		Actively listed		Admitted to hospital		Hospitalisation	
	*N* =	%	*N* =	%	*N* =	%	Total days	Mean days
Listing status in primary care								
Passively listed	39,430	32.0	–	–	2704	6.9	25,722	0.65
Actively listed	83,738	68.0	–	–	9099	10.9	97,968	1.17
Consultations, primary care								
0 or 1 consultation	95,810	77.8	59,077	61.7	7453	7.8	72,982	0.76
2 or 3 consultations	19,673	16.0	17,332	88.1	2661	13.5	29,699	1.51
4 or 5 consultations	5383	4.4	5108	94.9	1064	19.8	12,430	2.31
6 or 7 consultations	1522	1.2	1457	95.7	372	24.4	4622	3.04
8- consultations	781	0.6	765	97.9	253	32.4	3956	5.07
Type of practice								
A, private practice	20,428	16.6	15,798	77.3	1654	8.1	15,982	0.78
B, public practice	102,740	83.4	67,940	66.1	10,149	9.9	107,708	1.05
Sex								
Women	61,386	49.8	45,004	73.3	6447	10.5	67,729	1.10
Men	61,782	50.2	38,734	62.7	5356	8.7	55,961	0.91
Age groups								
16–19 years	6846	5.6	3777	55.2	182	2.7	2077	0.30
20–39 years	34,067	27.7	17,904	52.6	3571	10.5	25,571	0.75
40–59 years	39,374	32.0	26,176	66.5	2261	5.7	21,197	0.54
60–79 years	33,420	27.1	27,386	81.9	3646	10.9	45,225	1.35
80+ years	9461	7.7	8495	89.8	2143	22.7	29,620	3.13
Individual income								
First income quartile	29,588	24.0	18,843	63.7	2948	10.0	36,702	1.19
Second income quartile	30,933	25.1	23,764	76.8	4279	13.8	51,886	1.68
Third income quartile	31,339	25.4	21,415	68.3	2553	8.1	20,703	0.67
Fourth income quartile	31,308	25.4	19,716	63.0	2023	6.5	14,399	0.47
Educational level								
Less than 9 years	21,602	17.5	18,034	83.5	3173	14.7	41,848	1.94
Compulsory 9 years	15,956	13.0	10,128	63.5	1135	7.1	12,663	0.79
vCollege degree	54,693	44.4	37,319	68.2	4931	9.0	47,750	0.87
vUniversity degree	30,917	25.1	18,257	59.0	2564	8.3	21,429	0.69
Multimorbidity, all healthcare								
RUB 0	48,211	39.1	25,030	51.9	21	4.4	280	0.01
RUB 1	15,315	12.4	10,391	67.8	1224	8.0	5405	0.35
RUB 2	25,242	20.5	18,861	74.7	1603		11,149	0.44
RUB 3	30,566	24.8	25,983	85.0	6629	21.7	62,098	2.03
RUB 4	3233	2.6	2909	90.0	1849	57.2	29,134	9.01
RUB 5	601	0.5	564	93.8	477	79.4	15,622	26.03
Municipality								
A	49,931	40.5	27,700	55.5	5164	10.3	53,785	1.08
B	23,286	18.9	15,297	65.7	2269	9.7	24,044	1.03
C	25,405	20.6	21,723	85.5	2338	9.2	25,337	1.00
D	13,697	11.1	10,924	79.5	1107	8.1	11,819	0.86
E	10,849	8.8	8094	74.6	925	8.5	8705	0.80
Population	123,168	100.0	83,738	67.9	11,803	9.6	123,690	1.00

Unadjusted active listing on 31 December 2007 and hospitalisation during 2007 for the population of Blekinge > 15 years of age. *RUB* Resource Utilization Band; *Quartile* Four equally sized groups; *Municipality* Local government area

### Clustered zero-inflated negative binomial model

The zero-negative binomial model shows odds of any hospitalisation. Actively listed had OR 0.69 (95%CI 0.61–0.77) for any hospitalisation compared to passively listed. Having 6–7 consultations in primary care gave OR for any hospitalisation 0.60 (95%CI 0.48–0.72) compared to less than two consultations. Those listed in public primary care had OR 0.51 (95%CI 0.39–0.63) for any hospitalisation compared to those listed in private primary care (Table 2).

**Table 2:** Associations between active listing, consultations in primary care and hospitalisation for the population > 15 years of age in Blekinge in 2007 (N = 123,168), adjusting for sex, age, income, education and multimorbidity level

Multivariate binomial model	Odds for any hospitalisation		Mean days hospitalised	
	OR	(95%CI)	Days	(95%CI)
Listing status				
Passively listed	1.00		1.32**	(1.24–1.40)
Actively listed	0.69**	(0.61–0.77)	0.94**	(0.90–0.99)
Consultations, primary care				
0 or 1 consultation	1.00		1.21**	(1.13–1.29)
2 or 3 consultations	0.44**	(0.34–0.54)	0.80**	(0.75–0.84)
4 or 5 consultations	0.55**	(0.37–0.74)	0.77**	(0.72–0.83)
6 or 7 consultations	0.60**	(0.48–0.72)	0.77**	(0.66–0.87)
8 or 9 consultations	0.73*	(0.48–0.99)	0.80**	(0.51–1.10)
10- consultations	0.91	(0.63–1.19)	1.06**	(0.67–1.46)
Type of practice				
A, private practice	1.00		1.22**	(1.16–1.28)
B, public practice	0.51**	(0.39–0.63)	0.98**	(0.94–1.01)
Sex				
Women	1.00		0.94**	(0.89–0.98)
Men	1.13**	(1.09–1.17)	1.09**	(1.03–1.15)
Age				
16–19 years	1.00		0.63**	(0.37–0.90)
20–39 years	2.45**	(2.26–2.63)	1.12**	(1.01–1.22)
40–59 years	1.45**	(1.27–1.64)	0.77**	(0.69–0.86)
60–79 years	1.52**	(1.33–1.71)	0.91**	(0.85–0.98)
80+ years	2.05**	(1.88–2.21)	1.39**	(1.30–1.47)
Individual income				
First income quartile	1.00		1.10**	(1.08–1.13)
Second income quartile	1.10**	(1.06–1.14)	1.15**	(1.07–1.22)
Third income quartile	1.01	(0.84–1.04)	0.84**	(0.80–0.87)
Fourth income quartile	1.02	(0.97–1.08)	0.72**	(0.66–0.78)
Education level				
Less than 9 years	1.00		1.03**	(1.01–1.07)
Compulsory 9 years	0.89*	(0.79–1.00)	1.12**	(1.06–1.18)
College degree	0.87**	(0.61–0.93)	0.97**	(0.89–1.06)
University degree	0.88	(0.72–1.04)	0.97**	(0.93–1.01)
Multimorbidity level				
RUB 0	1.00		0.00*	(0.00–0.01)
RUB 1	6.66**	(5.95–7.37)	0.40**	(0.34–0.45)
RUB 2	6.50**	(5.78–7.23)	0.47**	(0.44–0.51)
RUB 3	8.13**	(7.49–8.77)	2.34**	(2.12–2.57)
RUB 4	9.85**	(9.21–10.49)	9.21**	(8.70–9.84)
RUB 5	11.13**	(10.66–11.60)	24.96**	(23.87–26.05)

*OR* Odds Ratio, *CI* Confidence Interval; * = *p* < 0.05, ** = *p* < 0.01; *RUB* Resource Utilization Band; Mean days hospitalised = average marginal effects combining both parts of the zero-inflated binomial model

**Fig. 1:**
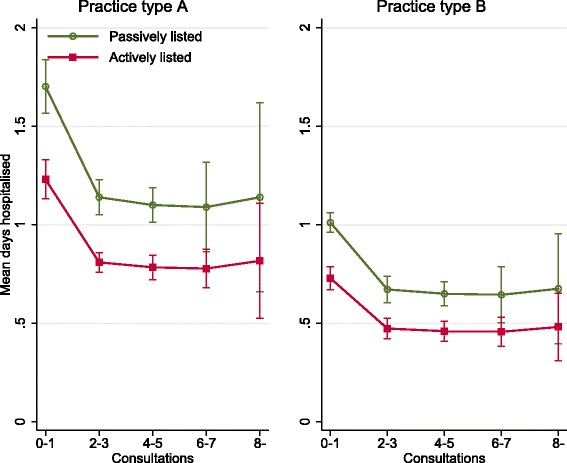
Predicted mean days hospitalised for the population > 15 years of age for listed in type A (privately owned practices with *N* = 20,428) and type B (publicly owned practices with *N* = 102,740) practices according to active listing and number of consultations, adjusting for sex, age, income, education and multimorbidity level

Mean number of days hospitalised for the entire population was calculated combining both parts of the multivariate model. Actively listed were in mean hospitalised for 0.94 (95%CI 0.90–0.99) days and passively listed 1.32 (95%CI 1.24–1.40) days. Patients with 0–1 consultations in primary care were in mean hospitalised 1.21 (95%CI 1.13–1.29) days, and with 6–7 consultations in primary care 0.77 (95%CI 0.66–0.87) days. Mean number of days hospitalised for those listed in private primary care was 1.22 days (95%CI 1.16–1.28) and for those listed in public primary care 0.98 days (95%CI 0.94–1.01) (Fig. 1, Table 2).

Setting active listing at 68% and number of consultations in primary care at 0.9 predicted totally 130,559 (95%CI 123589–146,273) days hospitalised. Comparing multivariate models showed that AIC for the logistic model including age, sex and multimorbidity level was 56,466. Including socioeconomic factors to this model gave LR-test 46 or relationship with primary care LR-test 850. AIC for a model including sex, age, multimorbidity level, socioeconomic factors and relationships with primary care was 55,587 (Table 3).

**Table 3:** Multivariate models on hospitalisation for the population > 15 years of age in Blekinge in 2007 (N = 123,168). Tests comparing multivariate models adjusting for relationships with primary care, sex, age, socioeconomic status and multimorbidity level

Model tests, multivariate models on hospitalisation	Area under ROC Curve		CV	AIC	LR test
	AUC	(95% CI)	%		
Sex and age	0.637	(0.632–0.642)	4.9	74,959	0
Adjusted for sex and age					0
Individual income and education	0.652	(0.647–0.658)	5.2	74,599	372
Relationships with primary care	0.668	(0.663–0.674)	6.2	73,638	1331
Multimorbidity level	0.858	(0.855–0.861)	13.7	56,466	18,503
Adjusted for sex, age and multimorbidity					0
Individual income and education	0.859	(0.856–0.862)	13.7	56,432	46
Relationships with primary care	0.864	(0.861–0.866)	14.1	55,626	850
Socioeconomy and relationship	0.864	(0.861–0.867)	14.2	55,587	901

N = 123,168; *AUC* Area Under the Curve; *CI* Confidence Interval; *CV* Coefficient of Variance; *AIC* Akaike’s Information Criterion; *LR test* Likelihood Ratio test

## Discussion

### Summary of main findings

Active listing is associated with reduced mean days hospitalised compared to passive listing, adjusting for socioeconomic status, multimorbidity level, sex and age.

Two or more consultations with a GP are associated with reduced mean days hospitalised compared to less than two, taking active listing, socioeconomic status and multimorbidity level into account.

Different odds of any hospitalisation give a difference in mean days hospitalised comparing private and public practices in primary care.

### Strengths and limitations of the study

We had the opportunity to combine healthcare data at individual level with socioeconomic data. Proxies of the relationship between patients and primary care, i.e. listing status and number of consultations, could be identified using patient records. We could analyse differences within primary care and adjust for sex, age, socioeconomic status and multimorbidity level. To our knowledge, this has not been done before. Our hypothesis was to find associations between hospitalisation and relationships with primary care, as well as differences within primary care.

Active listing and consultations in primary care could be regarded as measures of different aspects of the relationship between the population and primary care. Within this healthcare system, active listing could be regarded as patients acting to protect their relationship with a primary care practice. We expect active listing to underestimate relationships between patients and primary care, since they could be maintained by passive listing as well. Listing status was unlikely to be changed from active to passive during 2007 according to listing regulations. Data on listing by physician and consultations with staff members other than physicians were unreliable, hence not used. Number of consultations in primary care measures relationship as contacts between patients and primary care, but also includes morbidity burden, demand for healthcare services and availability of care. To account for these factors, we adjusted for sex, age socioeconomic status and multimorbidity level and clustered on municipality.

Primary care practices were grouped by ownership to estimate differences in efficiency within a primary care system. These groups had different characteristics regarding size and competence of the staff, number of listed patients and time since establishment. Further exploration of the impact of these characteristics was not possible. The established difference within primary care implicates that hospitalisation could be affected by differences in settings and processes between primary care practices although the regulations and funding are the same [13].

In Sweden, listing in primary care was introduced to empower patients and to introduce market models [18]. County councils regulate and organise local health care. Descriptions of health care in several European countries are available; and the listing system in Blekinge in 2007 is comparable to the Swedish system legislated in 2010 [18]. The European primary care monitor, describing primary care across Europe [24–26], facilitates comparisons within a European context.

We adjusted for morbidity using a summary measure of morbidity burden aiming to adjust for all cause multimorbidity. The aim was to study the association between relationships with primary care and all cause hospitalisation. Estimating morbidity burden from all health care adjusted for as much need for care, including need for secondary care, as possible. Other factors related to secondary care were not possible to adjust for, but the actual total hospitalisation was within lower 95%CI of the model prediction. Our use of diagnoses during year 2007 could underestimate multimorbidity for those hospitalised on December 31, if the diagnose was not registered before this admission.

Including socioeconomic status to adjust for demand for healthcare services and availability of care gave little improvement to the multivariate model. The cross-sectional design and choice of explanatory factors does not fulfil requirements of enough cause or causality, only of associations.

### Comparison with existing literature

Studies on larger populations, as countries, tend to show significant benefits of primary care, and there is evidence of regional and local variation in quality of care [27]. At county level, we confirmed significant benefits of active listing and 2–7 consultations in primary care on odds of any hospital admission and mean days hospitalised. Studies on predictors of high quality primary care have stated that longer consultations and good teamwork are important for quality of care, and also that no single type of practice has a monopoly on high quality care [28, 29]. Our study confirmed that more consultations in primary care reduced mean days hospitalised. We also found a difference between groups of primary care practices in mean days hospitalised, indicating a potential to improve the capability of primary care to reduce hospitalisation by modified settings and processes.

Hospitalisation has been studied using different concepts. An avoidable hospitalisation is one that could have been prevented by effective and available outpatient care, including primary care. Sets of ambulatory care sensitive disorders including chronic disorders as diabetes and asthma, as well as acute disorders, such as pneumonia or acute appendicitis has been used since decades [15, 16]. Most studies show lower hospitalisation rates for ambulatory care sensitive disorders in areas with greater access to primary care [15, 16]. Avoidable hospitalisations are often used as an indicator of primary care quality. However, the concept of avoidable hospitalisation does not address neither the complexity of the healthcare system nor the contribution of multimorbidity in individual patients. We showed that a summation measure of morbidity burden also could be used to study hospitalisation as an outcome of primary care. The multivariate model showed that increasing multimorbidity level was positively associated with both odds of any hospitalisation and mean days hospitalised (Table 2). When studying the association between relationships with primary care and hospitalisation we needed to address consultations for coexisting disorders. We also needed to understand the role of multimorbidity in use of healthcare resources [9, 17]. Within this healthcare system offering the same accessibility of primary care and adjusting for both socioeconomic status and morbidity burden we showed consistent difference according to relationship with primary care and also difference within primary care. The differences in mean days hospitalised between primary care practices aggregated from differences in odds of ever being hospitalised.

In a German study, associations between costs for hospitalisation and socioeconomic status were not found for the elderly [30]. Others have studied the associations between social deprivation, multimorbidity and hospitalisation and found complex associations, depending on settings [31, 32]. We found that morbidity burden and relationships with primary care had a stronger relation to mean days hospitalised than socioeconomic status for this population. We also found a difference related to primary care practices implicating that differences in structure and processes within primary care are associated with mean days hospitalised.

### Conclusions 

Hospitalisation could be analysed as an outcome of primary care. Good relationships with primary care, i.e. active listing and more consultations in primary care, are associated with reduced use of hospital care, taking sex, age, socioeconomic status and morbidity burden into account. To promote well performing primary care practices and their relationship with patients is an option to reduce hospitalisation, and failing to do so a risk of increasing hospitalisation.

The relationship between patients and primary care and how it is related to active listing and number of consultations needs further research. The relation between relationships with primary care and the outcomes of primary care also needs to be studied further. To study how different settings and processes worked to produce the differences we found between primary care practices could answer how to promote primary care to perform well.

To confirm our findings, more studies on hospitalisation as an outcome of primary care in other and larger populations including proxies of the relationship with primary care are needed. Different measures of multimorbidity needs to be compared when studying hospitalisation as outcome of primary care to conceptualise the differences between use of avoidable hospitalisation for sets of disorders and all cause hospitalisation using morbidity burden or patient complexity. The contribution of individual disorders to hospitalisation could also be studied comparing groups with the same need for care despite different patterns of disorders.

## Abbreviations

ACG: Adjusted Clinical Groups Case Mix System
GP: General practitioner
IRR: Incidence rate ratio
OR: Odds ratio
RUB: Resource utilization band
STATA: Stata Corporation, Texas, USA
